# A Case of Laceration of the Trachea

**Published:** 1911-10

**Authors:** W. L. Wallace, H. O. Brust

**Affiliations:** Syracuse, N. Y.; Syracuse, N. Y.


### A Case of Laceration of the Trachea

### Recovery Without the Usual Tracheotomy

By W. L. WALLACE, M.D., and H. O. BRUST, M.D.
Syracuse, N. Y.

ON June 13, 1910, Mr. Geo. M., a farmer, aet. 44 was received into the surgical ward of the Good Shepherd Hospital, Syracuse, N. Y. Six days previously while performing athletic stunts, he fell from a bar, striking the front of his neck across the back of a chair. He was at once in great distress for breath and coughed up much blood. For six days and six nights he was unable to lie down, and if he dropped off to sleep for a second, his breath would be instantly shut off. He also had great difficulty in swallowing any food or water.

When I saw him he was bent over the back of a chair holding his windpipe with his hand and his breaths were gasps. He was coughing up bloody sputum and was a terrible sight, suffering from dyspnoea, exhaustion and fear of impending death. His temperature was 99.6; his pulse, 100; respiration, 28, very labored. The front of his neck was dark red, swollen hard from chin to thorax. The neck was emphysematous, and all the tissues were distended with air as far down as his nipples. He was able to talk, and I therefore knew that the larynx was uninjured.

General anesthesia being out of the question, we operated, using one half per cent. of cocaine for the skin incision. With the patient sitting in a chair, we made a free cut from the cricoid cartilage to the sternal notch into the bloody, swollen, crackling tissues, and found the trachea crushed. Three or four of the rings were badly broken from about the third to the fifth or sixth. The proximal and distal ends of the broken trachea were entirely separated and out of line, and were held together only by the fibrous membrane which connects the posterior extremities of the cartilaginous hoops. We could not identify the isthmus of the thyroid which seemed to be torn apart.

We passed in a tracheotomy tube and gave him air. In a moment he coughed it out, and before it could be reintroduced he nearly smothered to death and we saved him only by getting the tube out of the way and holding the trachea open with a tenaculum. We then tried the experiment and found he could breathe easily, if we held the ends of the broken tube together. We therefore cut away the protruding splinters of the rings and sewed up the tracheal wound with fine chromic catgut. Most of the stitches were passed through the entire wall of the trachea, fastening and holding the broken rings to the sound rings above and below. As the mucous membrane tended to peel away from the rings and plug the breathing space, and as the broken rings tended to collapse, we found these through-and-through stitches necessary. Although we had never heard of its being done, we did not see why catgut passed through the mucous membrane should do any harm, when in so many cases a tracheotomy tube is so well tolerated. After getting the trachial tube airtight by stitching a piece of fascia over the repair, we were met with another difficulty. When we took off the tenacula, used to hold the ends of the trachea in line and together, the windpipe collapsed, lacking the supporting cartilage hoops. When we pulled forward again, he could breathe. We therefore passed two or three traction linen sutures into the front of the trachea and brought them forward out of the wound, keeping up the tension while the superficial wound was closed, and fastening them around the dressing covering the wound. A small rubber drainage tube was left at the lower extremity to take care of the infiltrated tissues.

The operation was easy and satisfactory, and was endured gladly, the patient leaving the operating room in good spirits and wonderfully relieved. He immediately took a drink of water, and lay down in his bed and slept naturally for many hours. The next day he was in fine condition and took liquid food easily. The drainage tube was gradually withdrawn during the next four or five days, and at the end of a week, the traction stitches were found to be no longer needed. They were therefore removed and the wound allowed to close, and he has had no further trouble. One month later, his breathing and speaking were normal.

Remarks:

Comminuted fractures of the trachea are usually compound, as the mucous membrane is generally lacerated. Fracture wounds of the trachea are usually treated by tracheotomy. The results of the treatment are very poor.

Can not the high mortality be reduced by other treatment? One case does not prove a method to be right, but this case is interesting because although it had stood six days and was in bad condition, it made an immediate recovery when simply sewed up. The method of passing the stitches through the entire wall of the trachea including the mucous membrane, was entirely satisfactory, and the traction stitches brought forward from a collapsing trachea and fastened into a wound might be used with advantage under other circumstances, as in some thyroidectomy cases.

